# Endokrine und neuroendokrine Tumoren

**DOI:** 10.1007/s00104-021-01512-8

**Published:** 2021-10-07

**Authors:** Philipp Riss, Katharina Scheuba, Oliver Strobel

**Affiliations:** Klinische Abteilung für Viszeralchirurgie, Medizinische Universität Wien, ENETS- Center of Excellence, Universitätsklinik für Allgemeinchirurgie, Währinger Gürtel 18-20, 1090 Wien, Österreich

**Keywords:** Gastroenteropankreatische neuroendokrine Tumoren, Nebennierentumor, Schilddrüsenkarzinom, Molekularpathologie, Molekulargenetik, Gastroenteropancreatic neuroendrocrine tumors, Adrenal tumor, Thyroid carcinoma, Molecular pathology, Molecular genetics

## Abstract

Endokrine Tumoren und hier im Speziellen neuroendokrine Neoplasien des Gastrointestinaltraktes (GEP-NETs), Phäochromozytome (PCs), Paragangliome (PGL) und Schilddrüsentumoren sind Paradebeispiele für die Bedeutung von Molekularpathologie und Molekularbiologie für Diagnostik, Klassifikation und letztendlich auch die (chirurgische) Therapie dieser Erkrankungen. Bei GEP-NETs erfolgt das Grading anhand des Ki-67-Index. Dieser bestimmt die Art der molekularen Bildgebung (DOTA [1,4,7,10-Tetraazacyclododecan‑1,4,7,10-tetraessigsäure]/DOPA [3,4-Dihydroxyphenylalanin]/FDG[Fluordesoxyglukose]-PET[Positronenemissionstomographie]/CT [Computertomographie]), die mögliche Therapie (chirurgisch und/oder Radiopeptidtherapie), antiproliferative und symptomkontrollierende Therapie mit Somatostatinanaloga und letztendlich auch die Prognose. PC/PGL können hereditär auftreten (MEN2A [multiple endokrine Neoplasie Typ 2A], VHL [Von-Hippel-Lindau-Tumorsuppressor], NF1 [Neurofibromatose Typ 1], SDH[Succinat-Dehydrogenase]-Mutationen), was die chirurgische Therapie und die präoperative Medikation maßgeblich beeinflusst. Die molekulare Bildgebung hat einen hohen Stellenwert und kann bei grenzwertiger Biochemie wegweisend sein. Auch Nebennierenrindenkarzinome können genetisch determiniert sein. Bei Schilddrüsentumoren ist v. a. die Pathologie der C‑Zelle (C-Zell-Hyperplasie, medulläres Schilddrüsenkarzinom) hervorzuheben. Bei hereditärer Erkrankung (FMTC [familiäres medulläres Schilddrüsenkarzinom], MEN[multiple endokrine Neoplasie]2) ist häufig eine frühe prophylaktische Operation notwendig und verhindert das Auftreten von fortgeschrittenen Karzinomen. Aber auch die Bestimmung des Resektionsausmaßes bei follikulären Läsionen bzw. die Unterscheidung zwischen „non-invasive follicular thyroid neoplasm with papillary-like nuclear features“ (NIFTPs) und follikulären Varianten des papillären Schilddrüsenkarzinoms kann mithilfe spezifischer Marker erfolgen. Insgesamt hat die Molekularpathologie eine zunehmende Bedeutung bei diesen Entitäten und ist auch Inhalt laufender Forschungsprojekte.

## Abstract (en)

Endocrine tumors and here in particular gastrointestinal neuroendocrine neoplasms (GEP-NET), pheochromocytomas (PC), paragangliomas (PGL) and thyroid tumors are prime examples of the importance of molecular pathology and molecular biology for the diagnostics, classification and ultimately also the (surgical) treatment of these diseases. The GEP-NETs are graded using the Ki-67 index. This determines the type of molecular imaging (DOTA/DOPA/FDG-PET/CT), the possible treatment (surgical and/or radiopeptide therapy), antiproliferative and symptom-controlling treatment with somatostatin analogues and ultimately also the prognosis. The PC/PGLs can be hereditary (MEN2A, VHL, NF1, SDH mutations), which significantly influences the surgical treatment and preoperative medication. Molecular imaging is very important and can lead the way in cases of borderline biochemistry. Adrenal carcinomas can also be genetically determined. In the case of thyroid tumors, the pathology of the C‑cell (C-cell hyperplasia, medullary thyroid carcinoma) should be emphasized. In the case of hereditary diseases (FMTC, MEN2), early prophylactic surgery is often necessary and prevents the occurrence of advanced carcinomas; however, the determination of the extent of resection in follicular lesions or the distinction between noninvasive follicular thyroid neoplasm with papillary-like nuclear features (NIFTP) and follicular variants of papillary thyroid carcinoma can also be determined with the help of specific markers. Overall, molecular pathology has an increasingly more important role in these entities and is also the topic of ongoing research projects.

Endokrine und neuroendokrine Neoplasien sind eine heterogene Gruppe von Tumoren verschiedener Organe, bei denen entsprechend ganz unterschiedliche Mechanismen in der molekularen Pathogenese relevant sind und für Diagnostik und Therapie ausgenutzt werden können.

Dabei sind im Gegensatz zu anderen Tumorerkrankungen oft bereits Zielmoleküle gut bekannt, und die Molekularpathologie ist bereits fester Bestandteil der klinischen Diagnostik und Therapie und gerade hier sehr relevant.

Der Beitrag konzentriert sich auf die Molekularpathologie von gastroenteropankreatischen neuroendokrinen Tumoren (GEP-NET) sowie Nebennieren- und Schilddrüsentumoren.

## Gastroenteropankreatische neuroendokrine Tumoren

Endokrine Tumoren des Gastrointestinaltraktes gehen von sehr unterschiedlichen Zelltypen des neuroendokrinen Systems aus (historisch auch APUD[„amino precursor uptake and decarboxylation“]-Zellen genannt). Gemeinsam haben sie, dass sie eine Vielzahl an biogenen Aminen und Peptiden sezernieren. NETs (neuroendokrine Tumoren) sind ein klassisches Beispiel für die Wichtigkeit von Molekularpathologie und Immunhistochemie (IHC), da diese bereits für die Diagnosestellung und dann für die Auswahl der weiteren diagnostischen und therapeutischen Verfahren unabdingbare Voraussetzung sind.

### Diagnostik der neuroendokrinen Tumoren

**Figure 1:**
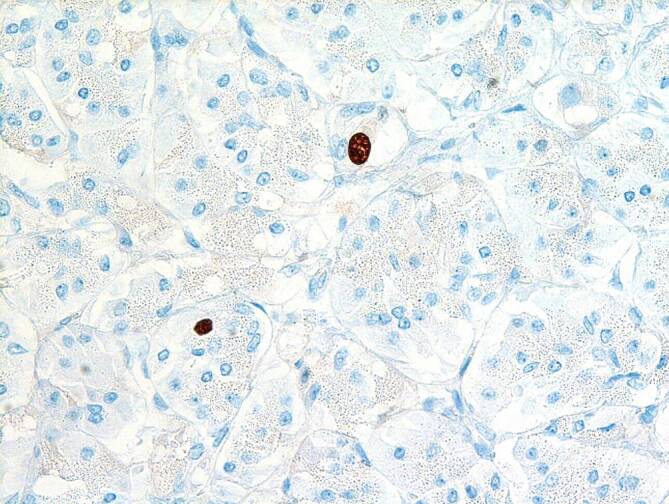

**Figure 2:**
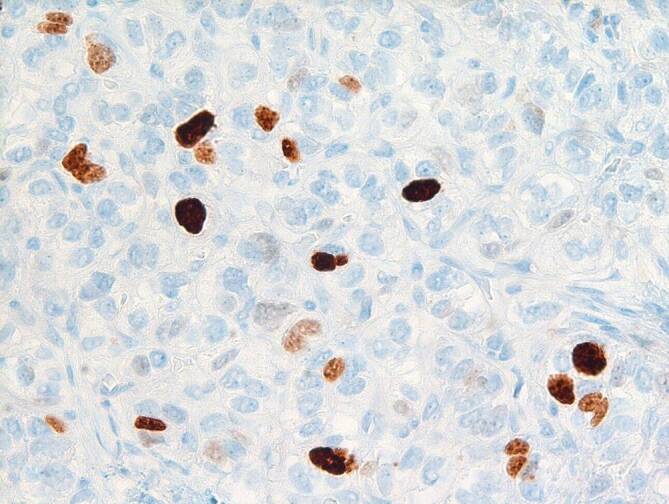

Für die Diagnose „NET“ müssen die Tumorzellen IHC-positiv für Chromogranin A (CgA) und/oder Synaptophysin sein. Für das Grading, das gerade bei GEP-NETs von großer Bedeutung ist, ist die Bestimmung des Ki-67-(MIB 1-)Index nötig. In Tab. 1 ist das aktuelle Grading-System dargestellt. Die Abb. 1 zeigt einen Dünndarm-NET G1 (Ki-67 < 2 %), Abb. 2 einen Dünndarm-NET G2 (Ki-67 3,5 %) (Bilder: Univ. Prof. Dr. P. Mazal, Klinisches Institut für Pathologie, Medizinische Universität Wien). Neben der Risikostratifizierung und Prognose bestimmt das Grading auch die weitere Diagnostik und Therapie. Die Bestimmung der exprimierten Somatostatinrezeptoren (v. a. SSTR2A) an der Zelloberfläche, die diagnostisch und therapeutisch relevant sein können, erfolgt ebenso mittels IHC.

**Table 1:** 

Grade	Ki-67-Index (%)	–
NET G1	≤ 2	DOTA-peptide-PET/CT^a^
NET G2	3–20	DOTA-peptide-PET/CT^a^
NET G3	> 20	DOTA-peptide^a^- oder FDG-PET/CT
NEC	> 20, schlecht differenziert	FDG-PET/CT

*NEC* neuroendokrines Karzinom, *DOTA* 1,4,7,10-Tetraazacyclododecan‑1,4,7,10-tetraessigsäure, *PET* Positronenemissionstomographie, *CT* Computertomographie, *FDG* Fluordesoxyglucose

^a^Alternativ: DOPA(3,4-Dihydroxyphenylalanin)-PET/CT

Die Bestimmung des Ki-67-Index ist bei NETs die Grundlage für die weitere Diagnostik und Therapie

Bei anhand des Zellbildes schlecht differenzierten G3-Tumoren spricht man von „neuroendokrinen Karzinomen“ (NEC), die in ihrer Aggressivität und Prognose dem kleinzelligen Bronchuskarzinom ähneln. Die selteneren, besser differenzierten NET G3 haben trotz eines hohen Ki-67-Index eine deutlich bessere Prognose und sind in Diagnose und Therapie den NET G1/G2 ähnlich [8]. Eine Unterscheidung kann auch molekularpathologisch getroffen werden mittels der Marker DAXX/ATRX (nicht bei NEC) und p53/pRb (exprimiert bei NEC) [23]. Dies ist jedoch derzeit noch keine Routinediagnostik.

CgA im Serum ist ein guter Tumormarker, der auch mit der Tumorlast korreliert und im Verlauf regelmäßig bestimmt wird. Er ist jedoch kein guter Screeningparameter und wird daher nur bei bereits diagnostiziertem NET empfohlen [6]. Neuronenspezifische Enolase im Serum (NSE) kann v. a. bei höhergradigen G2/G3-GEP-NET und v. a. G3-NEC als Tumormarker verwendet werden [21] und sollte zumindest einmal bestimmt werden [6]. Laborchemisch und klinisch muss auch auf eine mögliche Hormonproduktion hin untersucht werden. Genannt werden müssen hier unter anderem Gastrin, Insulin, vasoaktives intestinales Peptid (VIP) bei Pankreas-NETs und 5‑HIAA (5-Hydroxyindolessigsäure) (als Abbauprodukt von Serotonin) bei Dünndarm-NETs. Serotonin im Serum ist hingegen für die Diagnostik nicht geeignet. Die speziellen Aspekte der hormonproduzierenden NETs können hier nicht im Detail ausgeführt werden.

Die Leitlinien der Europäischen Gesellschaft für endokrine Tumore (ENETS) werden laufend aktualisiert und geben sehr differenzierte und evidenzbasierte Handlungsempfehlungen [22]. Seit Kurzem ist eine sehr aufwendig erstellte und übersichtliche deutschsprachige s2k-Leitlinie publiziert, die uneingeschränkt empfohlen werden kann [6].

Auf Basis des Gradings wird die weitere Diagnostik mittels molekularer Bildgebung durchgeführt. Dafür macht man sich die Expression von Somatostatinrezeptoren zunutze. „State of the art“ der molekularen Bildgebung bei G1/2-NETs (und NET G3 mit hohe SSTR[Somatostatinrezeptor]-Expression) ist die ^68^Ga-DOTA-Peptid-PET (Positronenemissionstomographie) in Kombination mit einer mehrphasigen CT (Computertomographie) oder auch MRT (Magnetresonanztomographie). Die auch noch durchgeführte Octreotid-Szintigraphie (Octreoscan) hat eine deutlich schlechtere Auflösung als die PET-CT oder PET-MRT. Alternativ kann auch der Dopaminstoffwechsel der Tumorzellen ausgenutzt werden (^18^F-DOPA[3,4-Dihydroxyphenylalanin]-PET-CT) [25]. Bei NEC G3 sollte aufgrund der schlechten Differenzierung der Zellen und des – aufgrund des sehr schnellen Wachstums – erhöhten Glukosestoffwechsels FDG als molekularer Tracer verwendet werden (ebenso bei NET G3 mit geringer SSTR-Dichte) [33].

Der Vollständigkeit halber soll erwähnt werden, dass, auch wenn dies per se nicht durch molekularpathologische Befunde beeinflusst wird, MRT der Leber und MRT sowie Endosonographie (ggf. mit Biopsie) des Pankreas zur Therapieplanung und/oder Verlaufskontrolle durchgeführt werden.

Somatostatinanaloga wirken antiproliferativ und blockieren die Hormonausschüttung

Bei jungen Patienten, bei multiplen Pankreas-NETs oder bei Patienten mit gleichzeitigem Hyperparathyreoidismus sollte das Vorliegen einer multiplen endokrinen Neoplasie (MEN) Typ 1 molekulargenetisch ausgeschlossen werden (Loss-of-function-Mutation im *MENIN*-Gen; 11q13) [13].

### Therapie neuroendokriner Tumoren

Neben der chirurgischen Resektion des Primarius, die in vielen Fällen auch bei kleinen Tumoren im metastasierten Stadium mit der Intention, lokale Komplikationen durch Tumorwachstum zu vermeiden, und zur Kontrolle hormonbedingter Symptome indiziert ist, gibt es eine Vielzahl an weiteren Therapieoptionen bei fortgeschrittenen NETs, und die Molekularpathologie hat auch hier einen wichtigen Stellenwert. Bei fortgeschrittenen NEC G3 ist die Chirurgie nur Ausnahmsweise Bestandteil einer multimodalen Therapie [8, 12].

Somatostatinanaloga (SSA; Octreotid, Lanreotid) binden in unterschiedlicher Affinität an die SSTR und hemmen einerseits die Hormonausschüttung, andererseits haben sie eine antiproliferative Wirkung. Die kurz wirksame Variante wird perioperativ verabreicht, um bei hormonaktiven Dünndarm-NETs eine starke Serotoninausschüttung zu verhindern und so eine Karzinoidkrise zu vermeiden. Auch die antiproliferative Wirkung ist lange bekannt, jedoch erst seit kürzer Zeit durch die Daten großer Studien (PROMID [24] für Octreotid, LAR und CLARINET [3] für Lanreotid) bestätigt und detaillierter in die Leitlinien aufgenommen. So werden SSA zur Kontrolle des Tumorwachstums bei langsam wachsenden GEP-NETs mit einem Ki-67 ≤ 10 % empfohlen [23].

### Peptid-Rezeptor-Radionuklid-Therapie

Die Peptid-Rezeptor-Radionuklid-Therapie (PRRT) macht sich die Tatsache zunutze, dass (wie beim ^68^Ga-DOTA-Peptid PET/CT) die Peptide an den SSTR binden. Der Betastrahler ^177^Lu hat sich als Standard etabliert [11] und kann bei dieser Therapie hochspezifisch direkt an die Tumorzelle gebracht werden. Die Resultate der NETTER-1-Studie [30] haben die Wirksamkeit deutlich gezeigt, und Langzeitdaten haben sie bestätigt [31]. Derzeit wird die PRRT in verschiedenen Therapieschemata bei fortgeschrittener Erkrankung – auch zur Symptomkontrolle – angewandt [23]. Voraussetzung für eine gute Wirksamkeit der PRRT ist eine starke Expression von SSTR auf den Tumorzellen. Die molekularpathologische Diagnostik hat hier also auch unmittelbaren Einfluss auf die Therapieauswahl.

Die Wirksamkeit der PRRT bei NET G3 wird gerade in der NETTER-2-Studie evaluiert [5].

Eine generelle Empfehlung zur adjuvanten Therapie bei NET G1/G2 kann nicht gegeben werden, da es keine prospektiv randomisierten Studien dazu gibt. Ausnahmen sind NEC G3 (Platin-basierte Chemotherapie), wobei auch hier Daten fehlen [23].

Everolimus, ein mTOR-Inhibitor, der auch als Immunsuppressivum in der Transplantationsmedizin zur Anwendung kommt, wird bei fortgeschrittenen pNET (neuroendokriner Tumor des Pankreas) G1/G2 eingesetzt, ebenso der Multikinaseinhibitor Sunitinib [23].

IFN(Interferon)-α, früher das Standardmedikament, gilt heute als Reserve, wenn andere Therapien nicht angezeigt sind oder nicht helfen (z. B. SSTR-negative Tumoren oder fortgeschrittene Dünndarm-NETs) [23].

## Nebennierentumoren

Tumoröse Veränderungen der Nebenniere(n) müssen immer auf eine mögliche Hormonaktivität abgeklärt werden. Sollten Hormone produziert werden (Katecholamine, Aldosteron, Cortisol, Geschlechtshormone oder ihre Vorstufen) oder besteht eine erhöhte Malignitätswahrscheinlichkeit, dann ist eine operative Entfernung indiziert [16]. Die Molekularpathologie hat v. a. bei Katecholamin produzierenden Tumoren – Phäochromozytom und extraadrenalen Paragangliomen (die hier mitbesprochen werden) – einen hohen Stellenwert sowie bedingt auch beim Nebennierenrindenkarzinom.

### Phäochromozytome und Paragangliome

Phäochromozytome (PC) stellen im deutschsprachigen Bereich die dritthäufigste Operationsindikation der Nebennieren dar [29]. Unterschied man früher benigne von malignen PC, so ist heute bekannt, dass auch vermeintlich „benigne“ PCs eine – wenn auch äußerst geringe – Metastasierungswahrscheinlichkeit haben [15]. Es wird daher bei PCs im Prinzip von einer malignen Erkrankung ausgegangen und von den Pathologen anhand des PASS-Scores [34] die Metastasierungswahrscheinlichkeit angegeben. „Benigne“ PCs wurden aus der WHO(Weltgesundheitsorganisation)-Nomenklatur 2017 gestrichen [15].

Bei Verdacht auf Phäochromozytom ist eine Biopsie absolut kontraindiziert

**Figure 3:**
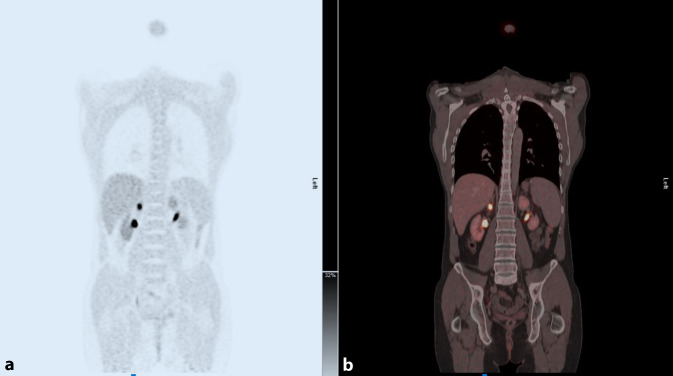

Bei Verdacht auf Phäochromozytom ist eine Biopsie absolut kontraindiziert. Die wichtigste Diagnostik bei Verdacht auf PC oder Paragangliom (PGL) ist die Bestimmung der Metanephrine im Serum (Sensitivität 89–100 %, Spezifität 79–97 %) [16]. Da diese jedoch einen weiten Graubereich haben, kann bei klinischem Verdacht eine funktionelle Bildgebung durchgeführt werden. Das ^18^F-DOPA-PET/CT hat hier eine sehr hohe Spezifität. Alternativ (z. B. bei bereits vorhandener Schichtbildgebung) wird eine (^123^I-)MIBG(Metaiodbenzylguanidin)-Szintigraphie durchgeführt. (^123^I-)MIBG kann auch bei fortgeschrittenem PC/PGL als Therapeutikum eingesetzt werden [1]. Es wirkt direkt in der Tumorzelle und kann als ein Betastrahler mit kurzer Reichweite diese zerstören. So kann das Fortschreiten der Erkrankung in vielen Fällen gebremst werden. Die Abb. 3 zeigt das ^18^F-DOPA-PET/CT eines jungen Patienten mit NF1 und Hypertonie. Die Metanephrine waren im niedrigen Graubereich. Das PET/CT konnte eindeutig ein rechtsseitiges PC zeigen, das retroperitoneoskopisch entfernt wurde (Bild: Univ. Prof. Dr. T. Traub-Weidinger, Univ. Klinik für Radiologie und Nuklearmedizin, Medizinische Universität Wien).

Bei Nachweis eines bilateralen PC sollte eine subtotale Adrenalektomie angestrebt werden

Bei Patienten mit unilateralem PC ohne Familienanamnese liegt in 12 % eine hereditäre Prädisposition vor, bei bilateralen PCs, PGL, jungen Patienten oder auffälliger Familienanamnese sind es über 30 %. Daher sollte die Klärung der Heredität mittels einer Genanalyse mit jedem Patienten mit einem PC oder einem PGL besprochen werden [16]. Gen-Panels werden derzeit nicht routinemäßig, sondern nur im Rahmen von Studien verwendet, werden aber sicherlich zukünftig einen größeren Stellenwert bekommen. Die wichtigsten Mutationen beim PC/PGL sind RET‑ protoonkogen Mutation, VHL (Von-Hippel-Lindau-Tumorsuppressor), NF1 (Neurofibromatose Typ 1) und SDH (Succinat-Dehydrogenase) (B/C/D), wobei das Vorhandensein einer hereditären Erkrankung bei der Therapieplanung eine große Rolle spielt. Bei sporadischen PCs wird eine unilaterale Adrenalektomie durchgeführt. Bei hereditärer Erkrankung mit hoher Wahrscheinlichkeit für ein (metachrones) kontralaterales PC ist hingegen die Planung einer subtotalen Adrenalektomie angezeigt. Es kann der Tumor entfernt und nach Möglichkeit ein Drittel (gesunder) Drüsenanteil erhalten werden, um eine spätere „totale Adrenalektomie“ mit Substitutionspflicht und Gefahr einer Addison-Krise zu vermeiden [16].

In Diskussion ist derzeit, ob Patienten mit PC/PGL immer eine präoperative Alphablockade benötigen oder auf diese in bestimmten Fällen verzichtet werden kann [27, 28]. So haben Patienten mit VHL- oder SDHB-Mutationen fast nie eine erhöhte Metanephrinausschüttung, und künftig könnte so anhand der Molekularpathologie über die Notwendigkeit einer präoperativen Alphablockade entschieden werden. In den rezenten deutschsprachigen Leitlinien wird dieses Thema diskutiert – derzeit wird eine Alphablockade jedoch weiterhin als Standard definiert [16].

### Nebennierenrindenkarzinom

Das Nebennierenrindenkarzinom („adrenocortical carcinoma“ [ACC]) ist ein seltener und sehr aggressiver Tumor. Meist haben die Tumoren bei Diagnose bereits eine Größe über 6 cm. Um ein ACC zu bestätigen oder eine Fernmetastasierung auszuschließen, sollte eine molekulare Bildgebung mittels FDG-PET/CT durchgeführt werden [9]. So kann auch die Abgrenzung zum gutartigen Nebennierenadenom erfolgen, das FDG negativ ist.

Die einzige Chance auf Heilung ist die frühzeitige chirurgische R0-Resektion des Primärtumors unter Einhaltung der chirurgisch-onkologischen Prinzipien – v. a. ohne Verletzung der Tumorkapsel [28]. Bei Tumoren < 6 cm kann dies auch bei entsprechender Expertise in bestimmten Fällen laparoskopisch erfolgen. Wichtig ist die Einhaltung der onkologischen Prinzipien einer En-bloc-Resektion und ggf. einer periadrenalen/renal hilären Lymphadenektomie [9], wobei bezüglich Lymphadenektomie die Datenlage schlecht ist und daher die CAEK[Chirurgische Arbeitsgemeinschaft Endokrinologie]-Empfehlungen diese nur bei Verdacht auf positive Lymphknoten empfehlen [16].

Eine molekulargenetische Analyse auf TP53(Tumor-Protein 53)- oder MMR[Missmatch-repair-Protein]-Mutationen sollte bei auffälliger Familienanamnese, speziell aber bei jungem Alter durchgeführt werden.

Die Einschätzung des Rezidivrisikos wird unter anderem mithilfe des Ki-67-Index durchgeführt (hohes Risiko bei Ki-67 > 10 %).

## Schilddrüsentumoren

Molekularpathologische Veränderungen bei Schilddrüsentumoren sind in großer Zahl beschrieben, und es werden laufend neue spezifische Mutationen bzw. Kombinationen an Veränderungen beschrieben. Trotzdem ist bei Schilddrüsentumoren die Molekularpathologie nur bedingt für Diagnostik und Therapie relevant, mit Ausnahme von medullären Schilddrüsenkarzinomen (und anderen C‑Zell-Pathologien), von NIFTPs („noninvasive follicular thyroid neoplasm with papillary-like nuclear features“) und der zielgerichteten Systemtherapie bei fortgeschrittenen Karzinomen. Auf diese Tumoren soll daher näher eingegangen werden.

### Medulläre Schilddrüsenkarzinome/C-Zell-Veränderungen

Calcitonin ist einer der am besten etablierten Tumormarker beim Menschen und hochspezifisch für das medulläre Schilddrüsenkarzinom (MTC). Es wurde bereits 2008 von Cheung et al. gezeigt, dass ein Calcitonin-Screening kosteneffektiv ist [4]. Die deutschsprachigen Leitlinien in ihrer aktualisierten Ausgabe [17] empfehlen (im Gegensatz zur ATA[American Thyroid Association]-Leitlinie [10]) vor jeder Schilddrüsenoperation die einmalige Bestimmung des Calcitoninwerts. So können C‑Zell-Veränderungen auch bereits in frühen Formen entdeckt werden. Die Höhe des basalen Calcitoninwerts lässt (bei Verwendung moderner Assays) Rückschlüsse auf den Fortschritt der Erkrankung zu und hilft, das Resektionsausmaß zu planen [20]. So gilt ein MTC als biochemisch gesichert bei Calcitoninwerten > 23 pg/ml (Frauen) und > 43 pg/ml (Männer). Eine laterale Lymphknotendissektion scheint erst ab 85 pg/ml (Frauen) und 100 pg/ml (Männer) notwendig zu sein, sofern der Tumor eine desmoplastische Stromareaktion zeigt. Ansonsten kann darauf verzichtet werden [19]. Etwa 25 % aller C‑Zell-Pathologien sind familiär und auf Mutationen im RET-Protoonkogen (MEN2A [multiple endokrine Neoplasie Typ 2A], FMTC [familiäres medulläres Schilddrüsenkarzinom], sehr selten MEN2B) zurückzuführen [18]. Die entsprechende molekularpathologische Diagnostik ist hier sehr wichtig, weil bei den Betroffenen sehr häufig – je nach Risikoprofil der aufgedeckten Mutation – schon im Kindesalter eine prophylaktische Thyreoidektomie und ggf. das Screening auf ein Phäochromozytom nötig werden. Detaillierte Empfehlungen wurden unter anderem von der Europäischen Gesellschaft für Endokrine Chirurgen (ESES) erarbeitet und publiziert [18].

Beim MTC steht mit dem (^18^F-)DOPA-PET/CT auch eine molekulare Bildgebung zur Verfügung, die bei fortgeschrittener Erkrankung hilfreich sein kann [2].

### „Noninvasive follicular thyroid neoplasm with papillary-like nuclear features“

„Noninvasive follicular thyroid neoplasm with papillary-like nuclear features“ (NIFTP) wurden erst vor Kurzem in die WHO-Klassifikation [26] aufgenommen und stellen eine benigne Form von follikulären Läsionen dar, die von Schilddrüsenkarzinomen (follikuläre Variante des PTC) abgegrenzt werden müssen. In der mittels Feinnadelaspiration gewonnenen Zytologie ist eine zuverlässige Differenzierung follikulärer Neoplasien nicht möglich. Hier kann – obwohl derzeit auch noch nicht standardmäßig eingesetzt – die Molekularpathologie zukünftig helfen, unnötige Thyreoidektomien und Lymphknotendissektionen sowie auch adjuvante Radiojodtherapien zu vermeiden [14]. Ob auch gänzlich auf eine operative Entfernung verzichtet werden kann, muss noch gezeigt werden. NIFTP weisen RAS(RAS-Protoonkogen)-Mutationen (sowie Mutationen in den *THADA*/*PPARG*-Genen) auf, aber im Gegensatz zu PTCs oder FTCs (follikuläre Schilddrüsenkarzinome) keine BRAF V600E, RET/PTC-Fusions- oder TERT(Telomerase-Gen)-Promoter-Mutationen [7, 14].

### Anaplastisches Schilddrüsenkarzinom

Anaplastische Schilddrüsenkarzinome (ATCs) sind hochaggressive Tumoren, die innerhalb kürzester Zeit zum Tod führen können. Eine aggressive Chemotherapie (oft in Kombination mit Strahlentherapie) kann hier deutlich lebensverlängernd sein [35]. Bei ATCs wurden zahlreiche Mutationen beschrieben, und so sind beim molekularpathologischen Nachweis der entsprechenden Mutationen zielgerichtete Therapien mit Dabrafenib (BRAF-Inhibitor) und Trametinib (MEK(MAP(„Mitogen-activated-protein“)-Proteinkinsasen)-Inhibitor) teilweise gute Therapieerfolge zu beobachten [32]. Aktuelle Leitlinien haben diese Therapien bereits aufgegriffen [7].

## Schlussfolgerung

Bei endokrinen Tumoren ist die Molekularpathologie seit Langem fester Bestandteil von Diagnose und Therapie. Speziell GEP-NETs sind hier besonders hervorzuheben, bei denen das gesamte chirurgische Management auf die molekularpathologische Klassifizierung aufbaut. Bei Nebennierentumoren kommt die molekularpathologische Diagnostik v. a. bei Phäochromozytomen und Paragangliomen, die – speziell bei hereditären Prädispositionen – ein diffiziles adaptiertes operatives Vorgehen erfordern, zum Einsatz. Bis auf MTC, bei denen die Therapie maßgeblich von den molekularpathologischen Ergebnissen bestimmt wird, hat die Molekularpathologie bei Schilddrüsentumoren v. a. in der Risikostratifizierung unklarer Knoten ihre Gewichtung und ist aktuell für das chirurgische Management noch wenig relevant. Ausnahme kann hier auch die präoperative NIFTP-Diagnostik sein, was jedoch – und das wird derzeit v. a. aus Kostengründen nicht flächendeckend durchgeführt – den konsequenten Einsatz kommerzieller Gen-Panels erfordern würde.

## Fazit für die Praxis

- Bei gastroenteropankreatischen neuroendokrinen Tumoren (GEP-NETs) bestimmt der Ki-67-Index das Grading, das die weitere Diagnostik und Therapie definiert.
- Standardbildgebung für NET (neuroendokriner Tumor) G1/G2 (NET G3) ist ^68^Ga-DOTA(1,4,7,10-Tetraazacyclododecan‑1,4,7,10-tetraessigsäure)-Peptid-PET(Positronenemissionstomographie)-CT (Computertomographie) oder -MRT (Magnetresonanztomographie).
- Bei NEC (neuroendokrines Karzinom) G3 und NET G3 mit hohem Ki-67-Index wird ein FDG(Fluordesoxyglucose)-PET-CT oder -MRT durchgeführt.
- Somatostatinanaloga wirken antiproliferativ und unterdrücken die Hormonausschüttung bei hormonaktiven NETs.
- Bei der PRRT (Peptid-Rezeptor-Radionuklid-Therapie) bindet das Radionuklid am SSTR (Somatostatinrezeptor) und bringt so den Betastrahler direkt an die NET-Zellen.
- Bei PC (Phäochromozytom)/PGL (Paragangliom) sollte eine molekulargenetische Analyse durchgeführt werden.
- DOPA(3,4-Dihydroxyphenylalanin)-PET/CT hat für PC/PGL eine hohe Spezifität.
- MIBG (Metaiodbenzylguanidin) kann für Diagnose bei PC und Therapie bei fortgeschrittener Erkrankung eingesetzt werden.
- FDG-PET/CT hilft, Nebennierenkarzinome von Adenomen zu unterscheiden.
- Bei ACC („adrenocortical carcinoma“) sollte bei jungen Patienten oder bei positiver Familienanamnese eine Mutationsanalyse (v. a. TP53 [Tumor-Protein 53]) durchgeführt werden.
- Bei erhöhtem Calcitonin und Verdacht auf C‑Zell-Pathologie sollte immer eine molekulargenetische Abklärung durchgeführt werden.

## Abkürzungen

5‑HIAA: 5‑Hydroxyindolessigsäure
ACC: Adrenocortical carcinoma
APUD: Amino precursor uptake and decarboxylation
ATA: American Thyroid Association
ATC: Anaplastisches Schilddrüsenkarzinom
CAEK: Chirurgische Arbeitsgemeinschaft Endokrinologie
CgA: Chromogranin A
CT: Computertomographie
DOPA: 3,4-Dihydroxyphenylalanin
DOTA: 1,4,7,10-Tetraazacyclododecan‑1,4,7,10-tetraessigsäure
ENETS: Europäische Gesellschaft für neuroendokrine Tumore
ESES: European Society of endocrine surgeons
FDG: Fluordesoxyglucose
FMTC: Familiäres medulläres Schilddrüsenkarzinom
FTC: Follikuläres Schilddrüsenkarzinom
GEP-NET: Gastroenteropankreatische neuroendokrine Tumoren
IFN: Interferon
IHC: Immunhistochemie
MEK: MAP(„mitogen-activated protein“)-Proteinkinasen
MEN: Multiple endokrine Neoplasie
MEN2A: Multiple endokrine Neoplasie Typ 2A
MIB 1: Antikörper gegen Ki-67
MIBG: Metaiodbenzylguanidin
MMR: Missmatch-repair-Protein
MRT: Magnetresonanztomographie
MTC: Medulläres Schilddrüsenkarzinom
NEC: Neuroendokrines Karzinom
NET: Neuroendokriner Tumor
NF1: Neurofibromatose Typ 1
NIFTP: „Non-invasive follicular thyroid neoplasm with papillary-like nuclear features“
NSE: Neuronenspezifische Enolase
PC: Phäochromozytom
PET: Positronenemissionstomographie
PGL: Paragangliom
pNET: Neuroendokriner Tumor des Pankreas
PRRT: Peptid-Rezeptor-Radionuklid-Therapie
RAS: RAS-Protoonkogen
RET: Rearranged during transfection
SDH: Succinat-Dehydrogenase
SDHB: Succinat-Dehydrogenase B
SSA: Somatostatinanalogon
SSTR: Somatostatinrezeptor
TERT: Telomerase-Gen
TP53: Tumor-Protein 53
VHL: Von-Hippel-Lindau-Tumorsuppressor
VIP: Vasoaktives intestinales Peptid
WHO: Weltgesundheitsorganisation
